# Obesity and Breast Cancer: A Paradoxical and Controversial Relationship Influenced by Menopausal Status

**DOI:** 10.3389/fonc.2021.705911

**Published:** 2021-08-13

**Authors:** Laura García-Estévez, Javier Cortés, Silvia Pérez, Isabel Calvo, Isabel Gallegos, Gema Moreno-Bueno

**Affiliations:** ^1^Breast Cancer Department, MD Anderson Cancer Center, Madrid, Spain; ^2^Centro de Investigación Biomédica en Red de Cáncer (CIBERONC), Madrid, Spain; ^3^International Breast Cancer Center (IBCC), Barcelona, Spain; ^4^Medical Scientia Innovation Research (MedSIR), Barcelona, Spain; ^5^Vall d’Hebron Institute of Oncology, Barcelona, Spain; ^6^Biochemistry Department, Universidad Autónoma de Madrid (UAM), Instituto de Investigaciones Biomédicas ‘Alberto Sols’ (CSIC-UAM), IdiPaz, & Centro de Investigación Biomédica en Red de Cáncer (CIBERONC), Madrid, Spain; ^7^MD Anderson International Foundation, Madrid, Spain

**Keywords:** overweight, menopause, estrogen, mammogram, adipocytokine, insulin, obesity

## Abstract

Breast cancer is the most common tumor in women worldwide, and an increasing public health concern. Knowledge of both protective and negative risk factors is essential for a better understanding of this heterogenous disease. We undertook a review of the recent literature and evaluated the relationship between obesity mediators and breast cancer development depending on menopausal status. Excess weight is now pandemic and has replaced tobacco as the main lifestyle-related risk factor for premature death. Although the prevalence of obesity/overweight has increased globally over the last 50 years, the potential harm attributable to excess fat has generally been underestimated. The relationship between overweight/obesity, breast cancer and overall risk appears to be highly dependent on menopausal status. Thus, obesity increases the risk of breast cancer in postmenopausal women but, conversely, it appears to be protective in premenopausal women. We evaluate the role of different clinical factors potentially involved in this seemingly contradictory relationship, including estrogen, mammogram density, adipokines, insulin-signaling pathway activation, and inflammatory status. A key focus of this review is to better understand the impact of body mass index and menopausal status on these clinical factors and, hence, provide some clarity into the inter-relationships involved in this controversial issue.

## Introduction

The incidence of obesity has nearly tripled since 1975 and it is now considered one of the worst pandemics of the 21th century. In 2016, more than 1.9 billion people aged over 18 years were considered to be overweight, and >650 million were obese; representing 39% and 13% of the adult global population, respectively (1). The prevalence of obesity varies widely by country; for example countries such as Vietnam (2.1%) and Japan (4.4%) reporting low rates compared with 37.3% in the United States and the highest rates in Oceania (Nauru, 61%; Cook Islands 55%; and Samoa/Tonga, both 46%) (1, 2). Except for some regions in sub-​Saharan Africa and Asia, more people are now obese than underweight (1); and projected trends for the next 10 years are disheartening, with an estimated 3.3 billion (58%) of the world adult population predicted to be overweight or obese by 2030 (3).

Obesity and overweight are generally associated with physical inactivity and a sedentary lifestyle. A survey of nearly 2 million participants in 2016 revealed that over a quarter (27.5%) of the global adult population failed to get enough physical activity with the highest prevalence (43%) in women, particularly in high-income countries (4). Such figures represent a setback for the global target set by the World Health Organisation (WHO) member states to reduce physical inactivity by 10% by 2025.

## Paradoxical Effects of Body Mass Index on Breast Cancer Risk in Relation to Menopausal Status

Overweight is defined as a body mass index (BMI >25 kg/m^2^, whereas obesity is categorised as a BMI >30 kg/m^2^ and is considered to be a chronic disease (5, 6). The association between breast cancer risk and excess weight (overweight/obese) varies according to menopausal status (**Table 1**), with independent studies reporting a negative correlation between obesity and risk in premenopausal women (9–11). Indeed, a pooled analysis of 7 studies including a total of 337,819 women (4,385 of whom had invasive breast cancer) reported a risk ratio (RR) of 0.54 [95% confidence interval (95% CI): 0.34-0.85] for breast cancer risk when comparing premenopausal women with BMI >31 kg/m^2^ versus those with a BMI of ≤21 kg/m^2^ (7). Likewise, a separate meta-analysis involving 9 studies also demonstrated an inverse correlation between obesity and per unit increase in BMI with regards breast cancer risk in premenopausal women [RR: 0.98; 95% CI: 0.97-0.99] (8). Similar findings were reported in an independent meta-analysis consisting of 34 datasets of women (a total of >2.5 million women) in which breast cancer risk was investigated relative to BMI (12). In total, breast cancer was reported in 7,930 premenopausal and 23,909 postmenopausal women and, overall, breast cancer risk was shown to be reduced by approximately 8% per 5 kg/m^2^ BMI increase in premenopausal women [RR: 0.92; 95% CI: 0.88-0.97; p=0.001]. In contrast, the risk was increased in postmenopausal women [RR: 1.12; 95% CI: 1.08-1.16; p<0.0001] (12). This latter meta-analysis undertaken by Renehan and colleagues (2008) included prospective cohort and large observational studies, and provides a stronger level of evidence than earlier case-control reports.

**Table 1:** Summary of large studies/analyses investigating the association of obesity with breast cancer (BC) according to menopausal status.

Reference	Study type	Comparative arms	Measure of association
Studies supporting a negative correlation between obesity and breast cancer risk in premenopausal women			
van den Brandt 2000 (7)	Pooled analysis [7 cohorts; 337,819 women and 4385 incident cases of invasive BC]	BMI >31 kg/m^2^ vs.≤ 21 kg/m^2^	RR: 0.54; 95% CI, 0.34-0.85
Bergstrom 2001 (8)	Meta-analysis [Premenopausal (17 studies); postmenopausal (27 studies)]	Unit increase in BMI	RR: 0.98; 95% CI, 0.97-0.99
Michels 2010 (9)	Prospective [113,130 premenopausal women]	BMI ≥30 kg/m^2^ vs. 20.0-22.4 kg/m^2^	HR: 0.81; 95%CI, 0.68-0.96
Berstad 2010 (10)	Case-control [2097 premenopausal women, 1900 postmenopausal women, and 4041 case controls]	BMI ≥35 kg/m^2^ vs. <25 kg/m^2^	OR: 0.81; 95%CI, 0.61-1.06
Harris 2011 (11)	Prospective [45,799 premenopausal women]	BMI ≥27.5 vs. <20.5*	HR: 0.74; 95%CI, 0.57-0.96
Renehan 2008 (12)	Meta-analysis [Premenopausal (20 studies); postmenopausal (31 studies)]	5 kg/m^2^ increase in BMI	RR: 0.92; 95% CI, 0.88-0.97
Premenopausal breast Cancer Collaborative Group 2018 (13)	Pooled analysis [19 cohorts: 758,592 premenopausal women]	5 kg/m^2^ difference in BMI	1.9- to 4.2-fold increased risk for lower BMI, depending on age
Studies supporting a positive association between obesity and breast cancer risk in postmenopausal women			
Rosenberg 2006 (14)	Population-based study [3345 postmenopausal women and 3455 matched controls]	≥30 kg vs. <10 kg weight gain	OR: 1.5; 95% CI, 1.2-1.8^a^
Reeves 2007 (15)	Prospective cohort study [1,222,630 women: Premenopausal BC 1179 cases, postmenopausal BC 5629 cases]	Obese women	RR: 1.29; 95% CI, 1.22-1.36
Renehan 2008 (12)	Meta-analysis [Premenopausal (20 studies); postmenopausal (31 studies)]	5 kg/m^2^ increase in BMI	RR: 1.12; 95% CI, 1.08-1.16
Neuhouser 2015 (16)	Extended follow-up from the WHI Clinical Trial. [67,142 postmenopausal women]	BMI >35 kg/m^2^	HR, 1.86; 95% CI, 1.60-2.17^a^

*Age adjusted; ^a^ER-positive patients.

HR, Hazard Ratio; OR, Odds Ratio; RR, Relative Risk; WHI, Women’s Health Initiative.

The largest, most recent study investigating the association of obesity with breast cancer risk came from The Premenopausal Breast Cancer Collaborative Group (13). This multicenter study used pooled individual-level data from 758,592 premenopausal women aged 18 to 54 years. Median follow-up was 9.3 years, during which time 13,082 cases of breast cancer were reported. The analysis revealed a significant 4.2-fold increased risk of breast cancer in underweight women versus women with obesity under 24 years, and a 1.9- to 2.5-fold increased risk in older patients. This led to an estimated 12% to 23% reduction in premenopausal breast cancer risk per 5 kg/m^2^ increase in BMI, depending on age. Moreover, estrogen-receptor (ER) and/or progesterone-receptor (PR) positive breast cancer was associated with BMI in all age groups, whereas hormone-receptor negative breast cancer was associated with BMI only in women aged 18-24 years. In this younger age group, the strength of the association between ER- and/or PR-receptor positive breast cancer and BMI (HR, 0.75; 95% CI, 0.70 – 0.81) was much stronger than that for ER- and PR-receptor negative breast cancer (HR, 0.85; 95% CI, 0.76 – 0.95). The lack of association of triple-negative breast cancer with BMI in women above 25 years-of-age was contrary to previous reports (17). The authors considered that the discrepancy possibly reflected differences in study design (case-controls versus prospective study); the former being more susceptible to potential selection bias.

Unlike premenopausal women, several studies and large meta-analyses have demonstrated a consistent, direct association between obesity and breast cancer risk in postmenopausal women. The Million Women Study followed 1.2 million UK women (45,037 with breast cancer) aged 50-64 years for a mean period of 5.4 years. The study identified an approximate 30% higher risk of breast cancer in postmenopausal women with obesity compared to women who were not obese (RR: 1.29; 95% CI: 1.22-1.36) (15). These findings are comparable to those of Renehan and colleagues reported above (postmenopausal breast cancer risk was positively associated with each 5 kg/m^2^ increase in BMI (RR: 1.12; p <0.0001) (12). The increased risk of breast cancer in postmenopausal women with obesity was greater in, and may be limited to, those not receiving menopausal hormone therapy. When factoring in hormone-receptor status, the association between BMI and postmenopausal breast cancer risk appeared to be restricted to ER-positive cases (14, 16).

## Potential Mechanisms that Could Explain the Paradoxical Relationship Between Obesity and Breast Cancer

Why obesity is a risk factor for breast cancer in postmenopausal women, but appears to be protective during premenopause is clearly an important question. A better understanding of the mechanisms involved could provide significant clues to the underlying pathophysiology and have clinical implications for breast cancer prevention and treatment. Mapping the precise cellular and molecular landscape of breast tissue in the context of an evolving physiology has shed light into potential driving factors responsible for breast cancer risk and progression; including the role of serum estrogens, leptin and adiponectin imbalances, mammogram density, insulin deregulation pathways and chronic inflammation.

### Difference in Estrogen Levels

Physiologically, estrogen production decreases during aging, with the natural consequence that postmenopausal women have lower blood levels of estrogenic hormones, such as estradiol, relative to premenopausal women. Estrogen synthesis in premenopausal women mainly localizes to the ovary, whereas in postmenopausal women estrogen production shifts to peripheral tissues. In postmenopausal women with obesity the main source of estrogen biosynthesis is adipose tissue.

The primary mediator of postmenopausal estrogen biosynthesis is aromatase (also known as CYP19A1, Cytochrome P450 Family 19 Subfamily A Member 1), an enzymatic complex found both in breast adipose and tumor tissues (18). Aromatase is responsible for the conversion of androgens produced by the adrenal cortex and the postmenopausal ovary into estrogens. This can lead to an increase in local estrogen levels by up to 10-fold in the endometrium and myometrium compared with that in breast tissue (19). Significantly, however, higher concentrations of estrogens were found in malignant breast tissue compared with non-malignant tissue.

Several meta-analyses of prospective studies have confirmed a relationship between increased serum estrogen levels and postmenopausal breast cancer (20–24). Although postmenopausal estradiol levels are lower overall, they have been shown to be higher in women with obesity compared with non-obese women (21 vs 12 pg/mL; with no overlap in 95% CI) (25). This contrasts with premenopausal data which found significantly lower estradiol levels (independent of age, race, and smoking) in women who were obese/overweight compared with women having normal bodyweight [32.8 pg/mL (95% CI: 30.6-35.2) vs. 39.8 pg/mL (95% CI: 37.0-42.8); p<0.001)] (25). Low estradiol levels in women with obesity may partly result from negative feedback in the hypothalamic-pituitary axis leading to a reduction in gonadotrophin secretion, and suppression of ovarian function and concomitant amenorrhea (26). Irregular menstruation and fewer ovulatory cycles may also result in a reduction in serum estradiol levels and this has been promulgated as contributory to the inverse association between obesity and breast cancer risk in premenopausal women (26).

### Mammogram Density

Mammographic breast density (MBD) is defined by the proportion of glandular to fatty tissue and is usually classified using a scoring classification proposed by the American College of Radiology (ACR BI-RADS^®^ Atlas, Breast Imaging Reporting and Data System) which describes 4 categories for breast density: a). almost entirely fatty; b). scattered fibroglandular tissue; c). heterogeneously dense fibroglandular tissue; and d). extremely dense fibroglandular tissue (27).

In women undergoing screening for breast cancer a high MBD poses two major challenges. Firstly, an elevated MBD may mask the presence of tumors within the dense tissue and diminish the detection sensitivity of screening mammography; and secondly, a high MBD is an independent risk factor for breast cancer (28). The magnitude of this association is 4.64-fold greater for the highest versus the lowest density categories. A low MBD associates with better breast cancer outcomes and lower risk of local recurrence without influencing the risk of metastasis or the mortality rates directly resulting from breast cancer. The strength of the association between MBD and breast cancer risk is greater than for most other established cancer risk factors, with the exception of age and some genetic factors (29).

The precise mechanisms underlying the positive association between elevated MBD and breast cancer risk remain poorly understood. It has been suggested that greater tissue density may be a consequence of increased epithelial cell concentrations, elevated levels of growth factors such as insulin-like growth factor-I (IGF-1), stromal fibrosis and/or epithelial hyperplasia; all of which represent potential breast cancer risk factors (28). Another plausible explanation is that there is higher aromatase expression in dense breast tissue compared to non-dense breast tissue (30). In addition, increased breast tissue density is associated with other well-established risk factors, including positive associations with hormonal replacement therapy, nulliparity, older age at first birth, and alcohol intake. Inverse associations have been linked to age, postmenopausal status and BMI (30).

In general, MBD correlates negatively with BMI; i.e., women who are obese or overweight have lower breast density than leaner women. The seemingly paradoxical relationship between obesity, breast density, and breast cancer risk lies in the fact that although adiposity has a positive correlation with absolute non-dense area and is a recognized risk factor for breast cancer, some reports indicate that absolute non-dense area correlates negatively with breast cancer risk (31). In this regard, Shieh et al. reported that elevated MBD was associated with an increased risk of ER-negative breast cancer independent of menopausal status or BMI in premenopausal women (32). Given that elevated breast density and overweight/obesity are both risk factors for ER-negative breast cancer, the authors hypothesize that they are likely to interact synergistically to augment the risk of ER-negative malignancy in younger women (32). In contrast, the study found no correlation between percent density and BMI in postmenopausal women. This result is consistent with the conclusions from the Nurses’ Health study, which reported an association between percent density and overall breast cancer in postmenopausal women, independent of BMI (33).

There is a tendency for MBD to decrease with age, while breast cancer incidence generally increases. This apparent contradiction fundamentally reflects that “true” breast tissue-age possibly results from the cumulative exposure to changing patterns of hormone and growth factor secretion and levels over time, rather than absolute age.

### The Role of Adipocytokines

Breast tissue is primarily composed of adipose and fibrous tissue, and mammary glands. Adipose tissue acts as an energy reservoir, but it is also responsible for the secretion of a variety of metabolites, hormones, and cytokines, collectively known as adipokines, and in this regard it can be considered as a complex endocrine organ (34). In humans, >100 adipocytokines have been described to date, including resistin, leptin, adiponectin, visfatin, hepatocyte growth factor, tumor necrosis factor-alpha (TNF-α), heparin-binding epidermal growth factor-like growth factor, interleukin-6 (IL-6) (35), and nerve growth factor (NGF) and brain-derived neurotrophic factor (36). Among these, adiponectin and leptin have received special attention as molecular mediators in the association between obesity and breast cancer (37).

Adiponectin is a 30 kDa monomer, also known as adipocyte complement-related protein (Acrp30), which circulates in the plasma as low- and high-molecular weight multimers (38). Plasma concentrations are relatively high for a hormone and are usually within the range 2–20 μg/ml. In women, adiponectin levels correlate negatively with body weight, BMI, body fat, and serum leptin concentrations (38). Adiponectin has multiple physiological actions, including modulation of the AMP-activated protein kinase (AMPK) axis by mechanisms involving liver kinase B1 gene (LKB1), and mediated *via* two adiponectin receptors, AdipoR1 and AdipoR2 (39). Activation of AdipoR1 and AdipoR2 induces the formation of homodimeric and heterodimeric complexes and triggers an AMPK-mediated cascade that regulates cell proliferation, apoptosis, angiogenesis and cellular metabolism. Activated AMPK monitors cellular ‘AMP: ATP’ (adenosine triphosphate) and adenosine diphosphate ‘(ADP): ATP’ ratios, offseting ATP consumption and maintaining energy homeostasis. AMPK regulates several downstream targets including enzymes involved in the synthesis of fatty acids and triglycerides such as acetyl-CoA carboxylase (ACC) and fatty acid synthase (FAS), transcription factors, and other regulatory proteins. Adiponectin also exhibits strong insulin sensitizing and balancing activity, and reduced adiponectin levels enhance insulin signalling associated with neoplasia. In addition, AMPK activation negatively regulates mitogen-activated protein kinase (MAPK), phosphatidylinositol 3−kinase (PI3K)/protein kinase B (Akt) (PI3K/Akt), WNT-β-catenin, nuclear factor kappa-light-chain-enhancer of activated B cells (NF-κB), and JAK2/STAT3 pathways (40). AMPK-mediated inhibition of mTOR (mammalian target of rapamycin) signaling results in disregulated cell proliferation, inhibition of gluconeogenic gene transcription, and induction of enzymes engaged with fatty acid oxidation (41). In breast cancer cells, adiponectin increases serine–threonine liver kinase B1 (LKB1) expression, which can negatively impact cell proliferation, increase apoptosis, decrease aromatase activity, an reduce invasive capacity (42).

Current data in support of a direct involvement of adiponectin in breast cancer is controversial. A meta-analysis by Gu et al. showed that serum adiponectin levels were lower in breast cancer patients irrespective of menopausal status; however, no information on BMI was collected (43). In contrast, two previous meta-analyses identified a significant association between adiponectin levels and breast cancer risk in postmenopausal women (44, 45). The most recent meta-analysis on this topic by Yoon et al. reported that low adinopectin and high leptin levels represent high-risk factors for breast cancer, particularly in postmenopausal patients (46). Assessment of the role of serum adiponectin in breast cancer highlights a trend linking elevated adiponectin levels with reduced breast cancer risk when a combined analysis involving pre- and postmenopausal women was performed. However, the results appear to be driven mainly by a stronger association in the postmenopausal group. Unfortunately, similar studies investigating an association between adiponectin levels and BMI in breast cancer are lacking.

Another adipokine, leptin, also represents a key molecular mediator in the relationship between obesity and breast cancer. Leptin is a hormone predominantly produced by adipocytes in healthy and malignant tissue and is overexpressed in individuals who are obese/overweight. Serum levels in humans usually range between 5–50 ng/ml, but in individuals with obesity, concentrations can exceed 100 ng/mL (47).

Leptin displays pleiotropic effects and regulates adipose and breast tissue cell proliferation. Upon binding to its receptor ObR, leptin activates JAK2/STAT3, MAPK, and PI3K/Akt signaling pathways to promote cell proliferation, hence acting as a growth-factor in cancer and involved in regulating tumor development, growth, and metastasis (48, 49). Leptin is also able to induce aromatase activity, thus potentiating ER signaling in breast cancer cells, and this may play a role in tumor progression (50). In addition, leptin can activate ObR-expressing macrophages and stimulate secretion of multiple pro-inflammatory and proangiogenic cytokines such as IL-1, TNF-α, IL-6, IL-11 and nitric oxide to modulate macrophage phenotype (anti-inflammatory M2 and pro-inflammatory M1). Some studies have also indicated that leptin and ObR are overexpressed in primary and metastatic invasive ductal breast carcinoma cells, compared with non-cancer mammary tissue (51).

Several studies strongly support the hypothesis that serum leptin levels correlate with breast cancer occurrence and tumor behavior (52, 53). To better clarify this issue Pan et al. conducted a meta-analysis of 35 case-control and cohort studies (54). Their results confirmed an association between leptin levels and breast cancer risk, and the authors outlined two key conclusions from this meta-analysis: a strong relationship between breast cancer risk and higher leptin levels in patients who were overweight/obese and, secondly, elevated leptin levels in some but not all the studies was associated with breast cancer risk in postmenopausal but not in premenopausal women. However, a high degree of heterogeneity was observed among the studies included in the meta-analysis.

### Insulin Signaling Pathway

Insulin is a peptide hormone synthesised by islet β-cells in the pancreas in response to elevations of glucose. Insulin regulates glucose uptake by muscle and adipose tissue and inhibits gluconeogenesis and glucose secretion by the liver. High insulin levels lead to an overload of glucose stores in liver and muscle. In addition to glucose homeostasis, insulin facilitates fatty acid transport, promotes lipogenesis, and inhibits lipolysis, thus contributing to the build-up of energy stores ready for mobilisation when insulin levels are low (e.g., during fasting) (55).

Under certain physiological and pathological conditions, cells may become less responsive to insulin and they eventually develop insulin resistance, causing both insulin and serum glucose levels to increase (3). Such outcomes may result from a combination of factors. For instance, endoplasmic reticulum stress, adiponectin reduction, leptin elevation, adipocyte death, macrophage infiltration and lipolysis can all contribute to the onset of chronic inflammation in obesity, and this is closely associated with marked insulin resistance and an increased likelihood of developing type 2 diabetes and other diseases (56, 57). A primary event in the transmission of inflammatory signals seems to be a reduction of functional insulin receptors and a blockage of insulin signaling pathways, and this has been hypothesized to lead to insulin resistance (58). Moreover, obesity may cause adipose tissue dysfunction, including secretion of abnormal levels of cytokines linked to insulin resistance, as well as impaired triglyceride storage and increased lipolysis. These abnormalities may in turn contribute to accumulation of non-esterified fatty acids (NEFAs) in the circulation and an overload of NEFAs by skeletal muscle and liver cells, which are liable to instigate decreased insulin responsiveness in these tissues. Increasing plasma NEFA levels might also contribute to a continuous loss of function of pancreatic β-cells, thereby causing inadequate insulin secretion (59).

When assessing type 2 diabetes as a risk factor for breast cancer the results appear to vary according to menopausal status. For example, a meta-analysis of 20 studies from nine different countries showed a 20% increase in breast cancer risk, independent of BMI, in postmenopausal women with diabetes compared with individuals who were not diabetic; significance was lost in premenopausal women (56). Likewise, a large prospective study by Michels et al. confirmed the positive relationship between type 2 diabetes and breast cancer risk (60). Interestingly, the study also found a negative trend between the two conditions in premenopausal women. Additionally, a positive association of plasma insulin and C-peptide with postmenopausal breast cancer risk has been reported, and this was independent of obesity status (at least in some studies) (56). In contrast, the situation in premenopausal women is unclear. The suggestion that relatively high circulating insulin levels may protect against breast cancer risk before the menopause is consistent with the apparently protective effect of diabetes observed by others authors; and it resembles closely the situation seen in obesity (61).

Insulin receptors are usually 6- to 10-fold overexpressed in breast cancer epithelial cells compared to healthy cells (62). Although primarily involved in the regulation of carbohydrate, lipid, and protein metabolism, insulin also acts as a growth factor, stimulating cell mitosis and migration, and inhibiting apoptosis (63). These effects may be potentiated under conditions of insulin resistance and concomitant impairment of insulin-regulated metabolic pathways. In general, insulin influences metabolic processes such as glucose transport through the PI3K pathway, whereas mitogenic stimuli involve the activation of Ras and the MAPK pathway.

### Chronic Inflammation

White adipocyte tissue (WAT) comprises the largest energy storage and the major endo- and paracrine organ in the human body. WAT consists of a variety of cell types (e.g., white adipocytes, immune cells, fibroblasts and endothelial cells) and these support tissue homeostasis and insulin sensitivity. In healthy-weight individuals, adipose tissue is well vascularized and the microenvironment is rich in anti-inflammatory cytokines such as IL-4, IL-10 and IL-13. WAT contains a variety of type 2 immune cells, including alternatively activated (M2-like) macrophages, group 2 innate lymphoid cells, type 2 T helper (TH2) cells and IL-4-producing eosinophils. Thus, in healthy individuals the adipose tissue molecular milieu is largely comprised of anti-inflammatory immune cells and cytokines (64). Weight gain causes adipocytes to become hypertrophic and die, and this is a key event in the change to an obese adipose tissue microenvironment which is proinflammatory. Adipocyte death triggers innate immune responses which shift the immune milieu towards a type 1 (pro-inflammatory) state which is associated with the infiltration of leukocytes, including macrophages, as well as CD8-positive T lymphocytes and mast cells (65). The rupture of the cell membrane also facilitates the release of cellular contents [e.g., lipids, cytokines and damage-associated molecular by-products (e.g. fatty acids, ATP, reactive oxygen species, cholesterol and nucleic acids)] into the microenvironment (66). Free fatty acids (FFAs) stimulate multiple inflammatory signaling pathways, and activation of transcription factor NF-κB a key mediator in the immune response and adipose inflammation (67). These signals enhance recruitment and accumulation of macrophages in situ. Up to 90% of macrophages in the obese adipose tissue microenvironment participate in the development of adipocyte hypertrophy by encircling the dying adipocyte forming crown-like structures (CLS); a hallmark of inflammation in adipose tissue (68). Macrophages are the main source of type 1 cytokines (e.g. TNF-α, IFNγ, IL-1β and IL-6) as well as pro-inflammatory immune cells (granulocytes, group 1 innate lymphoid cells, B cells and CD8+ T cells), which act by preserving a state of chronic inflammation.

Iyengar et al. conducted a study in patients undergoing mastectomy, as treatment for (n=211) or to reduce the risk of (n=26) breast cancer, and evaluated WAT inflammation as manifested by CLS-B (crown-like structures in the breast) (69). Overall, breast WAT inflammation was independent of BMI in postmenopausal women (p=0.008), with CLS-B present in 58/93 (62%) postmenopausal versus 64/144 (44%) premenopausal women. Moreover, BMI correlated with CLS-B positivity; specifically, CLS-B was present in 43/48 (90%) obese, 39/73 (53%) overweight, and 40/116 (34%) healthy-weight (BMI <25Kg/m^2^) individuals (p<0.001). The results remained statistically significant in a multivariate model after adjusting for age, menopausal status, and use of non-steroidal anti-inflammatory drugs (NSAIDs) or statins (p<0.001). The authors noted that WAT inflammation and the presence of CLS-B is associated with elevated tissue levels of proinflammatory mediators and aromatase (the rate-limiting enzyme for estrogen biosynthesis), and this provides a plausible explanation for the paradoxical observation that hormone-dependent tumors are more common during the age-related menopausal transition when circulating estrogen levels have declined (69).

**Figure 1:**
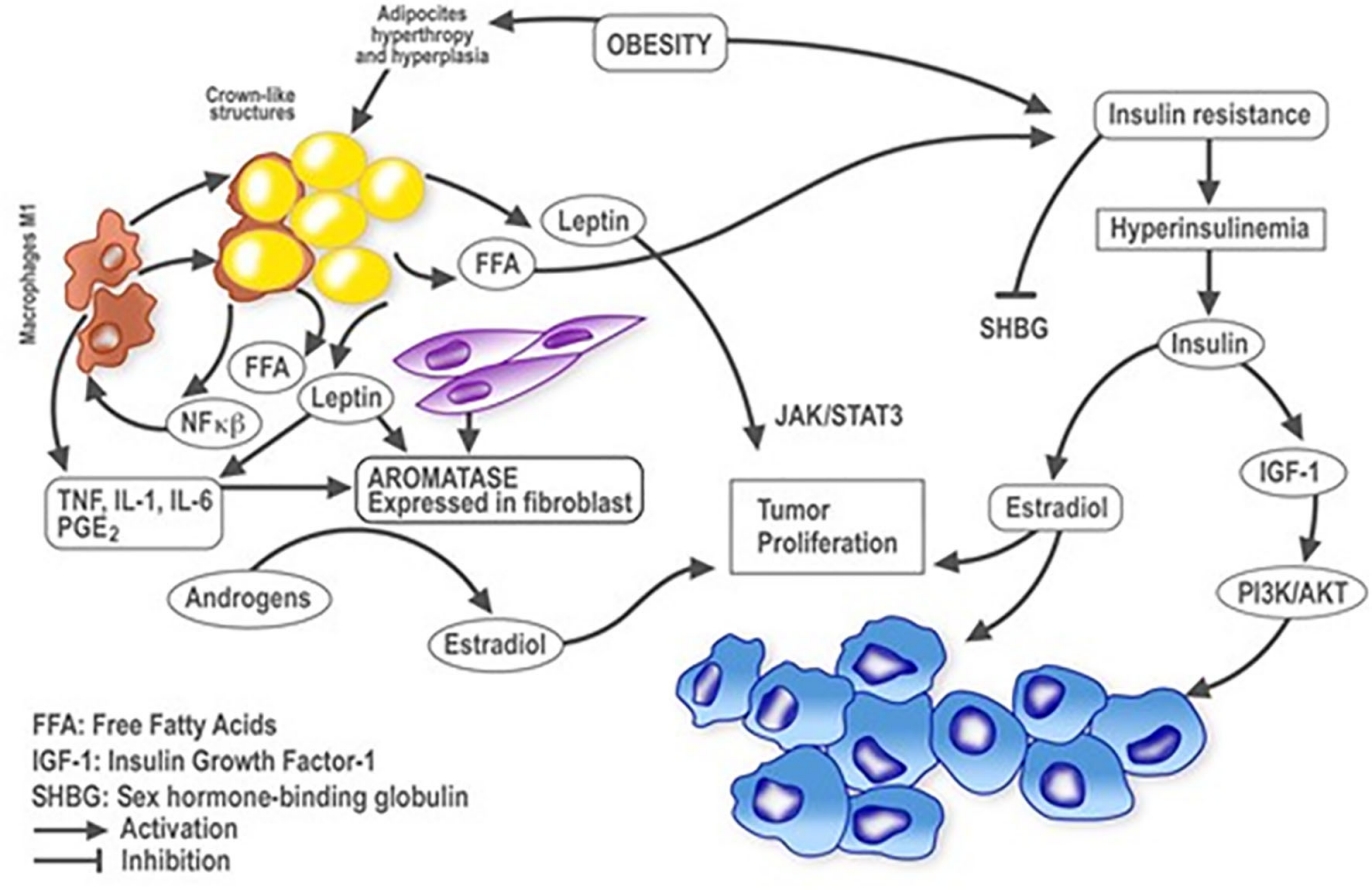
Plausible relationships between obesity and inflammation in breast cancer. Schematic representation of cellular and molecular events influenced by obesity and inflammation in the context of breast cancer development. Lines with arrowheads indicate activation; with flat ends, inhibition. NF-kB, nuclear factor kappa-light-chain-enhancer of activated B cells; TNFα, tumor necrosis factor alpha; IL, interleukin; PGE2, prostaglandin E2; FFA, free fatty acids; IGF-1, insulin growth factor 1; SHBG, sex hormone binding globulin.

Both adipocytes and macrophages release pro-inflammatory mediators associated with WAT inflammation such as TNF-α, IL-1β, IL-6 and COX-2–derived prostaglandin E2, which can upregulate aromatase expression (70). Aromatase is produced by mesenchymal cells and fibroblasts surrounding lipid-filled adipocytes, rather than in the adipocytes themselves. Aromatase utilises circulating androgens as a substrate to produce estrogens, mainly estradiol, which can diffuse into tissues, especially breast adipose tissue where it can enter the breast duct and stimulate epithelial cell proliferation (71). WAT inflammation is also associated with elevated circulating levels of leptin, a well-established inducer of aromatase (72). In addition, breast WAT inflammation is paralleled by increased tissue levels of aromatase (**Figure 1**) (70).

## Discussion

**Figure 2:**
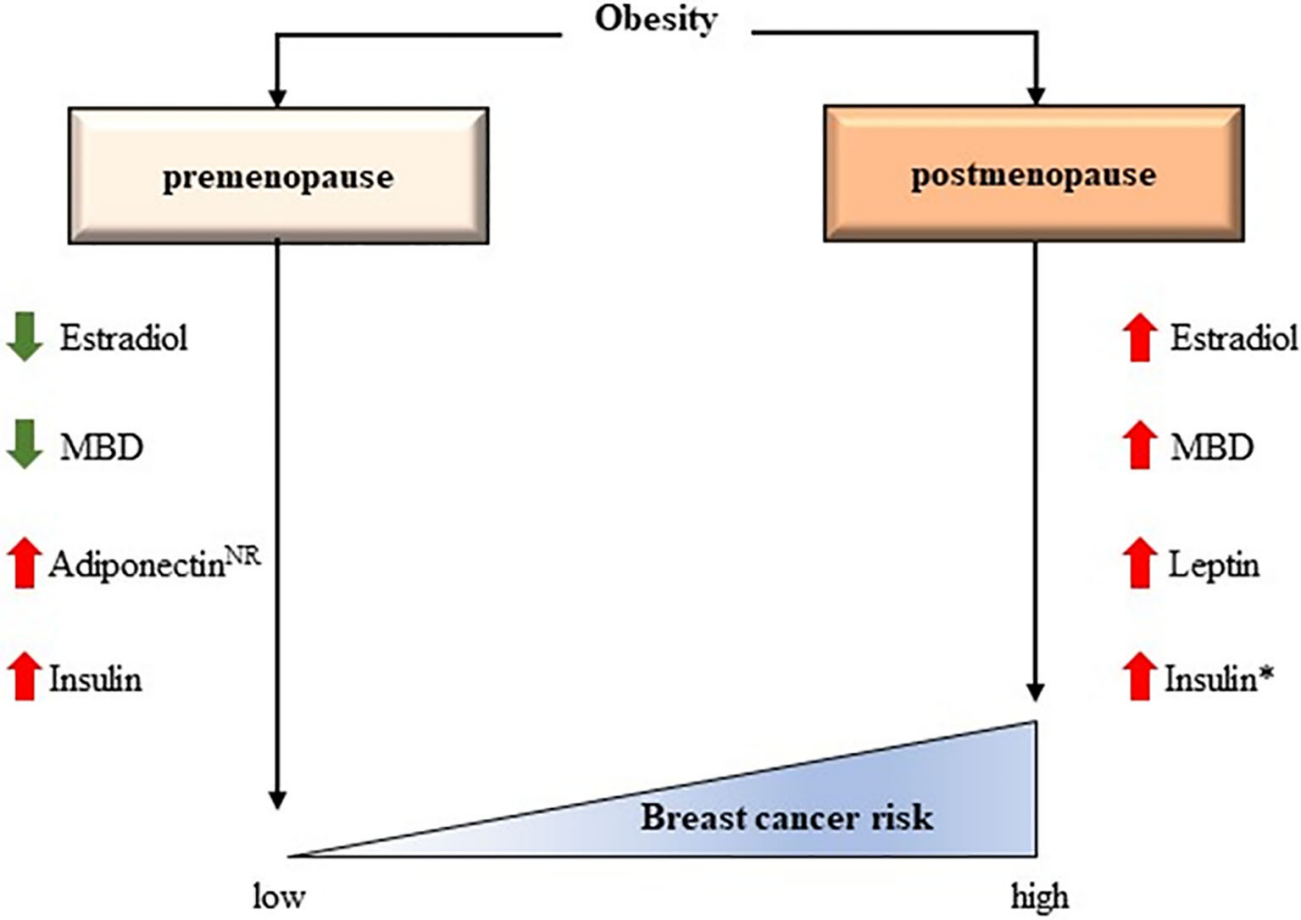
The controversial effects of obesity according to menopausal status. Some interactions have been confirmed while others have been hypothesized. MBD, Mammogram breast density; NR, Not clearly reported. *The effect of insulin in breast cancer risk could be independent of BMI (73).

The precise mechanisms whereby obesity plays a protective role against breast cancer in premenopausal women, but represents a risk factor after the menopause remain elusive (**Figure 2**) (74). Chronic inflammation clearly influences the physiology of the breast differently depending on menopausal state, suggesting a relevant role for estrogens and adipokine-driven signaling pathways.

It seems clear that the combination of oestrogens and obesity are able to protect against breast cancer, however the mechanism by which this interaction occurs is yet unknown. Zhao et al. conducted a study to address the impact of obesity in the breast according to menopausal status (75). Random fine needle aspirates (rFNAs) of breast tissue were collected from 57 premenopausal and 55 postmenopausal healthy women classified as normal-weight, overweight, and obese. Tissue samples were processed and subjected to TaqMan Low Density Array to determine the expression levels of 21 target genes. The study also measured breast tissue estradiol levels by liquid chromatography-tandem mass spectrometry and serum estradiol and follicle stimulating hormone (FSH) levels using radioimmunoassay and enzyme-linked immunosorbent assays, respectively. RPS6KB1 (an AMPK responsive gene for protein synthesis and cell growth), estrogen receptor α (encoded by the ESR1 gene) and its target gene GATA3, were significantly downregulated in rFNAs from premenopausal women with obesity. By contrast, levels of these biomarkers remained unchanged in relation to adiposity in postmenopausal women. On the other hand, prostaglandin-endoperoxide synthase 2 (PTGS2), cyclin D1 (CCND1), and TFF1 (an ESR1 target gene) were elevated in rFNAs from postmenopausal women with obesity. These results shed some light into mechanisms that potentially explain the different effects of obesity in the breast depending on menopausal status, hence contributing to our molecular understanding of breast biology in physiological and pathological situations.

Regarding adipokines, there exists a distinct relationship between leptin/adiponectin and breast cancer in postmenopausal women and such an association is far from clear in premenopausal women (73). According to Zhao and colleagues results, the protective role of obesity in premenopausal women could be favoured by activation of the AMPK signaling pathway and concomitant inhibition of proliferative stimuli *via* mTOR (75). Adiponectin may trigger activation of AMPK, which would explain the presence of high adiponectin levels in healthy, premenopausal women with obesity, but low levels in those with breast cancer. In relation to these observations, our group has initiated a prospective study in premenopausal women with and without breast cancer with a BMI of >25 kg/m2 (MDA-ADI-2020-01). The aim is to evaluate the blood levels of leptin and adiponectin between these two groups and to confirm the previously mentioned hypothesis: obese or overweight premenopausal women without breast cancer would have high levels of adiponectin and low levels of leptin despite a high BMI. By contrast, women with a high BMI and breast cancer would have high leptin levels and low adiponectin levels. If this hypothesis is confirmed, levels of adipokine and oestrogen in the blood could help in the prevention of breast cancer in premenopausal women.

In contrast to this hypothesis is the case-control study by Tworoger et al. showing a positive although non-significant association between adiponectin levels and breast cancer in premenopausal women; this suggests a differential role for the adipokine in breast tissue according to menopausal status (73).

## Conclusions

Obesity can have a marked impact in women since it is associated with an increased risk for many chronic conditions such as diabetes and heart disease, as well as it increases the risk of malignancies such as breast cancer. Whilst most evidence suggests an inverse relationship between obesity and breast cancer risk during premenopause, no clinical strategy could contemplate weight-gain as a therapeutic approach taking into consideration the complications associated with excess fat and the risk of perpetuating a state of overweight through menopausal transition and into the menopause itself. The metabolic and cardiovascular risks associated with excessive weight would undoubtedly exceed any benefits achieved for breast cancer. Of greater importance is to research and fully understand the complex interplay between the various metabolic and hormonal factors involved, since this could potentially help define possible alternative treatment strategies. In an obese environment, the relationship between estrogens and adipokines and the activation of specific signaling pathways clearly differs between menopausal stages, and this is an area where future research can help determine the molecular/biochemical processes involved. Ultimately, a better knowledge of how obesity and adipose tissue inflammation interact with female sex hormones, and how these changes affect the pathogenesis of breast cancer, should enable us to develop new therapeutic strategies to reduce breast cancer risk and improve patient outcomes in individuals who are obese.

## Author Contributions 

LG-E and GM-B designed and wrote the manuscript. All authors provided editorial support and read the manuscript. All authors contributed to the article and approved the submitted version.

## Funding

This research did not receive any specific grant from funding agencies in the public, commercial, or not-for-profit sectors.

## Conflict of Interest

The authors declare that the research was conducted in the absence of any commercial or financial relationships that could be construed as a potential conflict of interest.

## Publisher’s Note

All claims expressed in this article are solely those of the authors and do not necessarily represent those of their affiliated organizations, or those of the publisher, the editors and the reviewers. Any product that may be evaluated in this article, or claim that may be made by its manufacturer, is not guaranteed or endorsed by the publisher.
